# A type II phosphatidylinositol-4-kinase coordinates sorting of cargo polarizing by endocytic recycling

**DOI:** 10.1038/s42003-024-06553-3

**Published:** 2024-07-12

**Authors:** Anezia Kourkoulou, Olga Martzoukou, Reinhard Fischer, Sotiris Amillis

**Affiliations:** 1 National and Kapodistrian University of Athens, Department of Biology, Athens, Hellas Greece; 2 Karlsruhe Institute of Technology - South Campus, Institute for Applied Biosciences, Department of Microbiology, Karlsruhe, Germany

**Keywords:** Golgi, Fungal biology

## Abstract

Depending on their phosphorylation status, derivatives of phosphatidylinositol play important roles in vesicle identity, recognition and intracellular trafficking processes. In eukaryotic cells, phosphatidylinositol-4 phosphate pools generated by specific kinases are key determinants of the conventional secretion pathways. Earlier work in yeast has classified phosphatidylinositol-4 kinases in two types, Stt4p and Pik1p belonging to type III and Lsb6p to type II, with distinct cellular localizations and functions. Eurotiomycetes appear to lack Pik1p homologues. In *Aspergillus nidulans*, unlike homologues in other fungi, AnLsb6 is associated to late Golgi membranes and when heterologously overexpressed, it compensates for the thermosensitive phenotype in a *Saccharomyces cerevisiae pik1* mutant, whereas its depletion leads to disorganization of Golgi-associated PH^OSBP^-labelled membranes, that tend to aggregate dependent on functional Rab5 GTPases. Evidence provided herein, indicates that the single type II phosphatidylinositol-4 kinase AnLsb6 is the main contributor for decorating secretory vesicles with relevant phosphatidylinositol-phosphate species, which navigate essential cargoes following the route of apical polarization via endocytic recycling.

## Abstract (web summary)

The Golgi-associated phosphatidylinositol-4-kinase AnLsb6 in *Aspergillus nidulans* may have adopted functions of the missing Pik1p homologue in Eurotiomycetes.

## Introduction

Compartmentalization is an indispensable feature of eukaryotic cells, aiming to bind and distinguish internal membranous structures physically and functionally. Central roles in this process play the phosphorylated species of the phospholipid phosphatidylinositol (PtdIns), the so-called phosphoinositides (PtdInsPs), acting as key determinants of membrane identity in both endocytic and exocytic routes, also directing signal transduction, vesicular budding and fusion, cytoskeleton dynamics, regulation of integral plasma membrane proteins, endosome dynamics and Golgi functions^1,2^. The various known species of PtdInsPs are generated from ER-originating PtdIns that are transported intracellularly through membrane contact sites and undergo reversible phosphorylation and dephosphorylation, implicating specific PtdIns-kinases and phosphatases^3^. Roughly, the phosphorylation status at positions 3, 4 and 5 of the inositol rings and their combinations define the known species of PtdInsP, the identity and functionality of which is further supervised and secured by the coincidence and concerted action with other accessory proteins and regulators (e.g. coat proteins, Rab GTPases, other effectors etc.)^4,5^. Based on the predominant subcellular localization of PtdInsP species, it is generally accepted that PtdIns-4 phosphates (PtdIns4P) associate with Golgi-mediated secretion pathways, PtdIns4,5P_2_ are enriched at the plasma membrane, whereas PtdIns3-phosphates are represented in endocytosis-related routes, however, exceptions and emerging roles are constantly reported, highlighting the complexity comprising these phospholipid pools^2,6–13^.

Most abundant in cells are PtdIns4P and PtdIns4,5P_2_, displaying however significant ratio variations with other PtdInsP species^3^. Concerning the generation of PtdIns4P in yeasts, two families of PI4Ks can be distinguished based on their general enzymatic properties^1,3^. Type III members in *Saccharomyces cerevisiae* are represented by Stt4p and Pik1p (homologous to mammalian PI4KIIIα and PI4KIIIβ), their function being non-redundant, and they have been shown to generate physiologically discrete pools of PtdIns4-phosphates at the Golgi and at the plasma membrane^14^. Stt4p, existing as a heterodimer with its regulatory partner Ypp1, is mostly a plasma membrane-associated protein recruited by Efr3 and Sfk1 and is involved in the synthesis and supply of PtdIns4P at the plasma membrane and ER membranes, overall regulating phosphatidylserine and sphingolipid homoeostasis, actin dynamics and MAPK pathway activation^14–16^. Pik1p localizes to the nucleus and also to the Golgi via interaction with the frequenin orthologue Frq1 and regulates trafficking at the late secretory level through interaction with established Golgi-associated partners, like the GTPase Arf1p and its guanine nucleotide exchange factor Sec7p^17,18^, while also playing additional and distinct roles in the generation of PtdIns4P in the nucleus upon nutrient starvation^19^. In *Schizosaccharomyces pombe*, Pik1 and Stt4 have been implicated in septation and cell division, and in these cases Stt4 appears to be recruited to the plasma membrane by the Efr3 homologue, whereas in the case of Pik1 the interaction with the Frq1 homologue Ncs1 does not appear to regulate Golgi localization^20–22^. Overall, functions of the type III members seem to be conserved in higher eukaryotes, including regulation of Golgi organization, scaffolding, vesicle exit through coat protein recruitment and autophagy^2,8,23,24^. Lsb6p encodes for the single type II PtdIns4-kinase in *S. cerevisiae*, but its function remains poorly characterized, nevertheless being implicated in actin assembly and actin-dependent endosome motility during endocytosis^25–29^. In mammalian cells, two type II PtdIns4-kinases (PI4KIIα and PI4KIIβ) have been described to associate with the plasma membrane and with the Golgi, as well as with endosomes and ER-PM contact sites, indicating that besides operating at a plasma membrane supply with PtdIns4P, they might also be involved in recycling, as well as in selective and non-selective autophagic pathways^3,7,30^. In plant cells, PtdIns4P is generated also by at least two subfamilies of PtdIns4-kinases, PI4Kα and PI4Kβ, localized at the plasma membrane and the Golgi, interestingly however, the PtdIns4P pool is shifted towards the plasma membrane and much less in the trans Golgi, making many proteins that were traditionally associated with PtdIns4,5P_2_ in other organisms now rely on PtdIns4P^9,31^.

In filamentous fungi the information on the function of phosphoinositide kinases is scarce^32,33^. Exceptions are the PtdIns4,5P_2_-related Mss4p-homologue of *Neurospora crassa* that is required for apical tip growth^34^, the PtdIns3,5P_2_-related Fab1p-homologue that affects vacuolar-cellular homeostasis and aflatoxin biosynthesis in *Aspergillus flavus*^35^, and the endosome/vacuole associated Lsb6p-homologue in *Fusarium graminearum* that is involved in endocytosis and regulates PtdIns4P pools on endosomes^36^.

The current study focuses on the role of the single type II PtdIns4-kinase homologue in the intracellular trafficking pathways of the filamentous ascomycete *A. nidulans*, making use of targeted genetic approaches combined with fluorescent molecular marker imaging. Results presented herein should advance knowledge and prove useful beyond this fungus, in aspects related to biotechnological applications and suppression of pathogenic species, as well as in biomedical applications, where several diseases have their origins in defective phosphoinositide kinases and phosphatases^2,24,37–41^.

## Results

### Identification and subcellular localization of the type II PtdIns 4-kinase homologue, AnLsb6

**Fig. 1:**
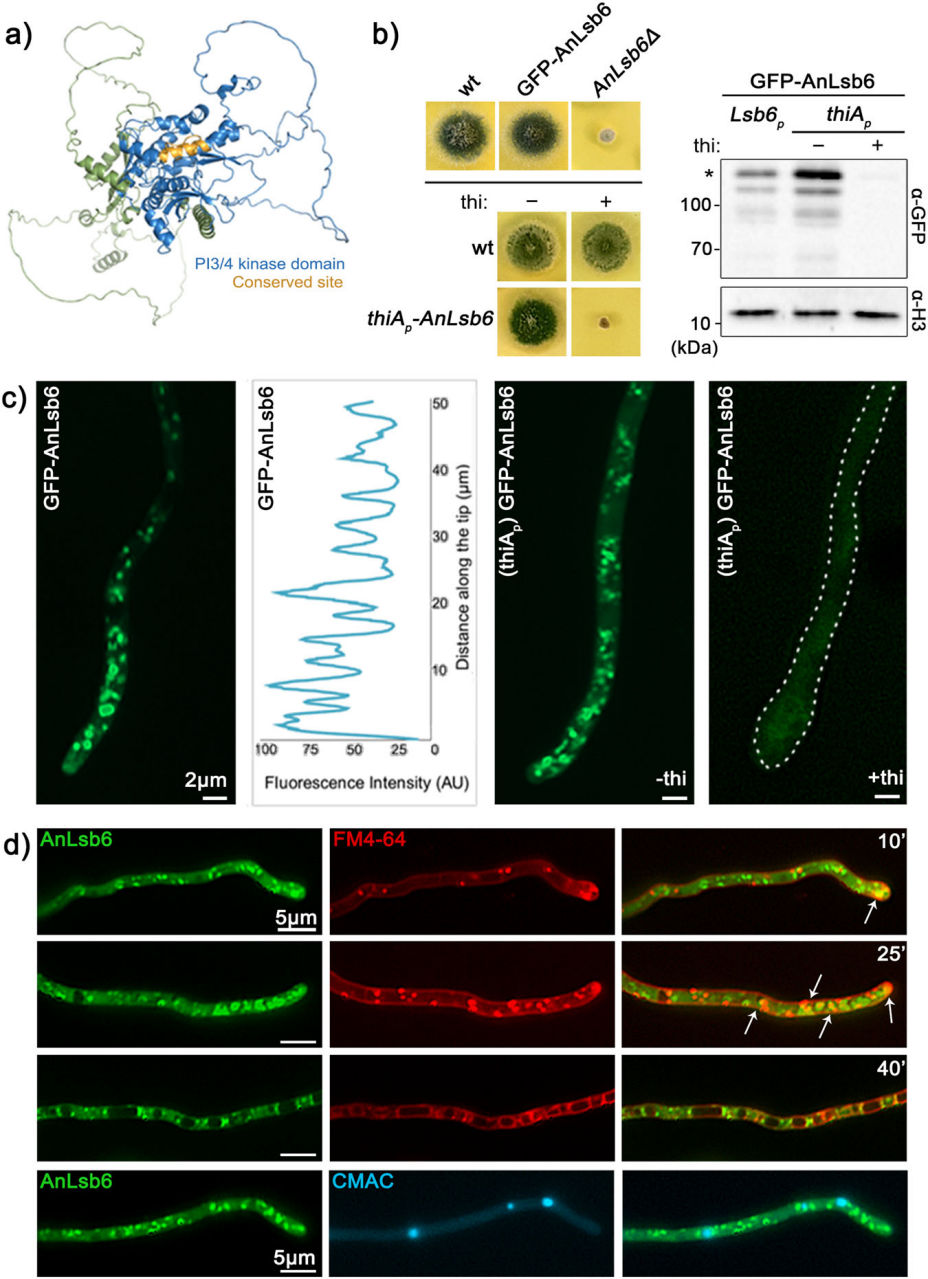
AnLsb6 is essential for growth and localizes in polarly distributed structures. **a** Model of AnLsb6 generated with AlphaFold2. The structure is colored based on domain and conserved site topology, as predicted by InterPro analysis. For coloring based on Predicted Aligned Error (PAE), see Supplementary Fig. 1. The conserved site corresponds to residues ^385^ELEKLVILDYIMRNTDRGLDN^405^, part of which (^397^RNTDRGLDN^405^) is predicted to operate as a catalytic loop (PROSITE entry PS00916). **b** Growth of strains carrying deletions of AnLsb6 ORF (*ANlsb6Δ*), N-terminal GFP-tagged version of AnLsb6 and isogenic strains carrying in locus thi-repressible alleles of *thiAp-AnLsb6* in the absence (−) or presence (+) of thiamine (thi). Growth was assessed on minimal media, pH 6.8, 37^o^ C for 72 h. Western blot analysis comparing protein levels of GFP-AnLsb6 and GFP-AnLsb6 expressed under the *thiAp* promoter in the absence (−) or presence (+) of thiamine (thi). Equal loading is indicated by Histone 3 levels. An asterisk (*) indicates the intact GFP-AnLsb6 protein. Comparison of N- and C-terminal GFP-tagged versions of AnLsb6 and uncropped images of the blots are shown in Supplementary Fig. 2. **c** Localization of strains expressing N- and C-terminal tagged versions of AnLsb6. A strain repressed for GFP-AnLsb6 expression ( + thi, right panel) is also shown as a control of thiamine repression. Raw data on the quantification of fluorescent intensity are given in Supplementary Data 1. **d** Localization of GFP-AnLsb6 in the presence of FM4-64, which dynamically labels endocytic steps (plasma membrane, early and late endosomes, vacuoles) and in the presence of the vacuolar stain 7-amino-4-chloromethylcoumarin (Blue CMAC). A minor overlap is observed at earlier time points of the FM4-64 chase (indicated by arrows), in agreement with the non-association with CMAC, that mostly stains terminal endocytic stages.

Two families of PtdIns 4-kinases can be distinguished based on their general enzymatic properties, the type II (*S. cerevisiae* Lsb6p, mammalian PI4KIIα and PI4KIIβ) and the type III (*S. cerevisiae* Stt4p and Pik1p, mammalian PI4KIIIα and PI4KIIIβ)^1,3^. Most interestingly, members of the Eurotiomycetes class appear to lack Pik1p homologues (JGI MycoCosm), proteins which are essential in both *S. pombe* and *S. cerevisiae*. BlastP searches in *A. nidulans* revealed however single homologues of Stt4p and Lsb6p, called AnStt4 and AnLsb6^32^. We have decided to focus on the role of the single type II PtdIns 4-kinase in *A. nidulans*, AnLsb6 (AN10791), a 775 aa protein displaying a characteristic phosphatidylinositol 3- and 4-kinase catalytic domain profile with conserved residues, further reflected in the generated AlphaFold2 structural model (Interpro tool; Family IPR039756, domain IPR000403, conserved site IPR018936; and Supplementary Fig. 1) illustrated in Fig. 1a. AnLsb6, however lacks other typical features, like proline-rich domains, frequennin-binding domains, or cysteine-rich palmitoylation sequences^3^.

Indeed, mutants carrying a deletion of the entire *AnLsb6* ORF, despite displaying a deleterious phenotype, could be propagated as genetically stable haploids (Fig. 1b). Nevertheless, and also in order to facilitate other downstream applications, like generation of biomass or genetic crossings, an approach making use of the thiamine repressible promoter (*thiA*) was chosen as an alternative^42^. Under these conditions and driven by this promoter, although expression levels appeared to be slightly higher compared to the native *AnLsb6p*, the protein could be efficiently depleted in the presence of thiamine in the culture medium (Fig. 1b and Supplementary Fig. 2). Concerning the subcellular localization of AnLsb6, the C- or N-terminally tagged GFP versions resulted in distinct, rather immotile foci, characterized also by a progressive accumulation towards the hyphal tip (Fig. 1c). Ring-like and cisternae-like structures, that in some cases also have the tendency to associate with each other, were also observed in the N-terminal tagged GFP-AnLsb6, whereas in the C-terminal tagged AnLsb6-GFP a more scattered fluorescence signal and a swollen hyphal tip was evident. The latter, along the fact that only the N-terminal tagged GFP-AnLsb6 fully complemented an AnLsb6 deficient strain (Fig. 1c and Supplementary Fig. 2a), suggests that a C-terminal GFP tag interferes with proper protein folding or generally affects interactions in trans. An effective repression in a strain driving N-terminal tagged GFP-AnLsb6 with the *thiA* promoter is also demonstrated by the reduction of the fluorescence signal (Fig. 1c and Supplementary Data 1). Furthermore, in a first attempt to define the nature of the observed polarized fluorescent signal, the subcellular localization of GFP-AnLsb6 was followed in the presence of FM4-64, which dynamically labels endocytic steps upon progressive incubation, and in the presence of CMAC that labels acidified late vacuolar compartments^43,44^. GFP-AnLsb6 is associated with endosomal structures to a slight extent, mainly at earlier endocytic stages and much less with terminal endocytic compartments, or vacuoles (Fig. 1d).

### AnLsb6 associates with Golgi membranes

**Fig. 2:**
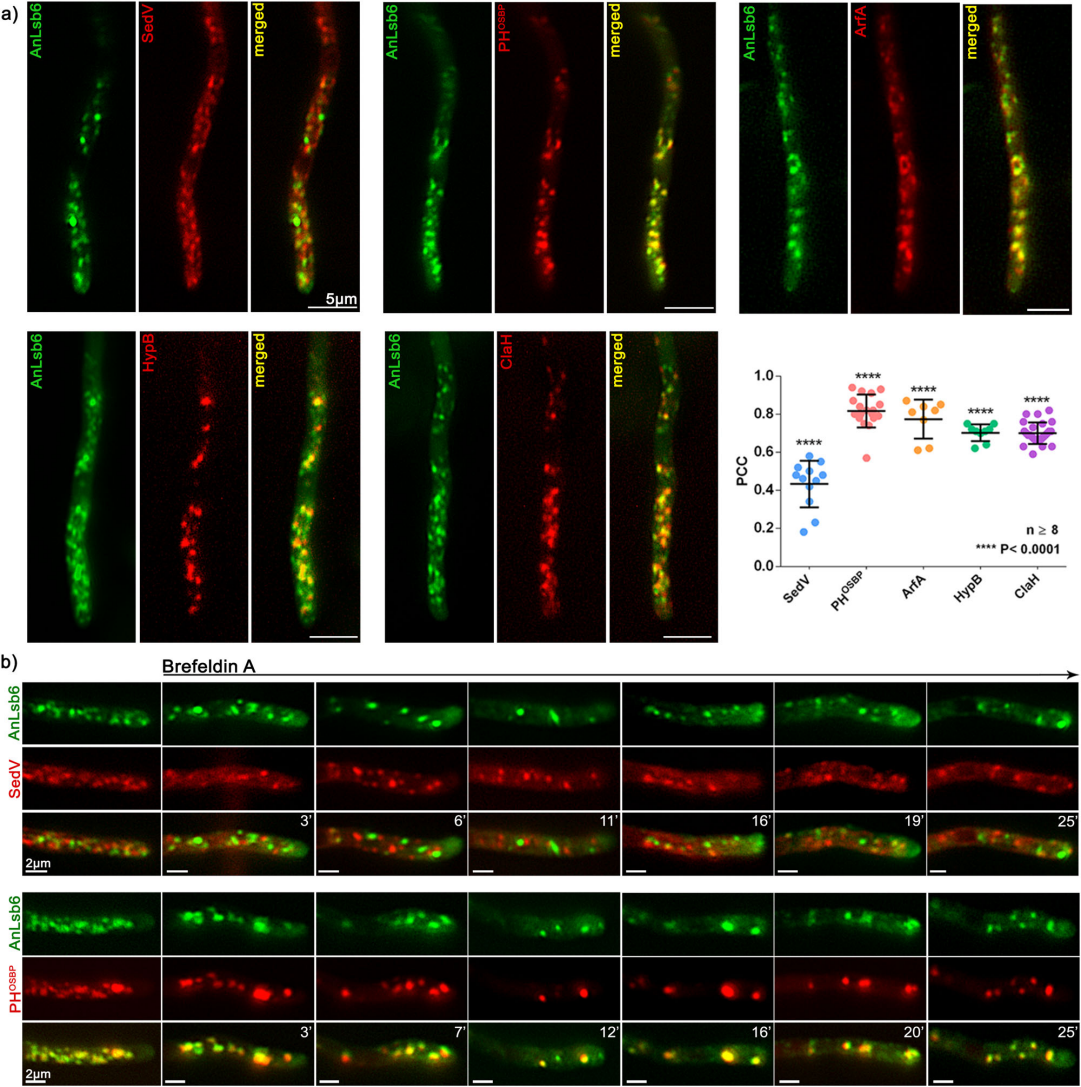
AnLsb6 associates with Golgi structures. **a** Localization of GFP-AnLsb6 compared to mRFP- or mCherry-tagged early- and late-Golgi markers. AnLsb6 associates significantly with late-Golgi markers like the pleckstrin homology domain of the human oxysterol binding protein PH^OSBP^ (PCC = 0.84, *P* < 0.0001, *n* = 18), GTPase ArfA^ARF1^ (PCC = 0.78, *P* < 0.0001, *n* = 8), GEF HypB^SEC7^ (PCC = 0.72, *P* < 0.0001, *n* = 9) and clathrin heavy chain ClaH (PCC = 0.71, *P* < 0.0001, *n* = 28), but much less with the early Golgi t-SNARE SedV^SED5^ (PCC = 0.43, *P* < 0.0001, *n* = 12). Raw data on the quantification of co-localization by calculating Pearson’s Correlation Coefficient (PCC) are given in Supplementary Data 2. See Methods for statistical analysis and statistical tests used. Error bars represent Standard Deviation (SD). **b** Representative images in different hyphae of the subcellular localization of AnLsb6 with SedV^SED5^ or the pleckstrin homology domain of the human oxysterol binding protein PH^OSBP^ in the presence of the inhibitor Brefeldin A, chased for a time period of 25 min. Notice that a fraction of Brefeldin bodies primarily includes PH^OSBP^ and not SedV^SED5^.

The subcellular localization of AnLsb6 shows the characteristic polarized distribution of vesicular structures (Golgi cisternae), resembling localization of other characterized Golgi-associated proteins in *A. nidulans*^45,46^. For this reason, colocalization was attempted using available established markers for the early and late Golgi compartments, such as the early Golgi t-SNARE SedV^Sed5^, the pleckstrin homology domain of the oxysterol binding protein (PH^OSBP^) binding on Golgi PtdIns4P enriched membranes (see also later), the Ras GTPase ArfA^ARF1^ involved in Golgi organization and secretion^47,48^, the HypB^SEC7^ Arf1 GEF^45,49,50^, or the clathrin heavy chain ClaH coating secretory vesicles at the post Golgi^51,52^. This analysis showed that whereas less colocalization is observed with the early Golgi marker SedV^SED5^ ^53^, a significant association of GFP-AnLsb6 is observed with all other late Golgi markers (Fig. 2a and Supplementary Data 2). This association with later Golgi stages is further supported by chase experiments in the presence of the Golgi inhibitor Brefeldin A, where preferentially GFP-AnLsb6 and PH^OSBP^ tend to colocalize in transiently collapsing Brefeldin bodies (Fig. 2b)^52–54^, by the mis-localization of GFP-AnLsb6 towards the hyphal apex when the trans-Golgi Arf1 GEF HypB^SEC7^ is depleted, by the strong dis-organization and appearance of cytoplasmic haze when clathrin heavy chain is depleted^52,53,55^, the latter also suggesting that GFP-AnLsb6 may also be operating more downstream at the late/post Golgi stage (Supplementary Fig. 3a), and finally by the effective reconstitution of bimolecular fluorescence (BiFC) in strains carrying AnLsb6 and HypB^SEC7^ tagged with N-terminal YFP_N_ and C-terminal YFP_C_, respectively, indicating at least close proximity of these two proteins (Supplementary Fig. 3b).

### AnLsb6p affects PtdIns4P-containing membranes and rescues a Pik1p mutant

**Fig. 3:**
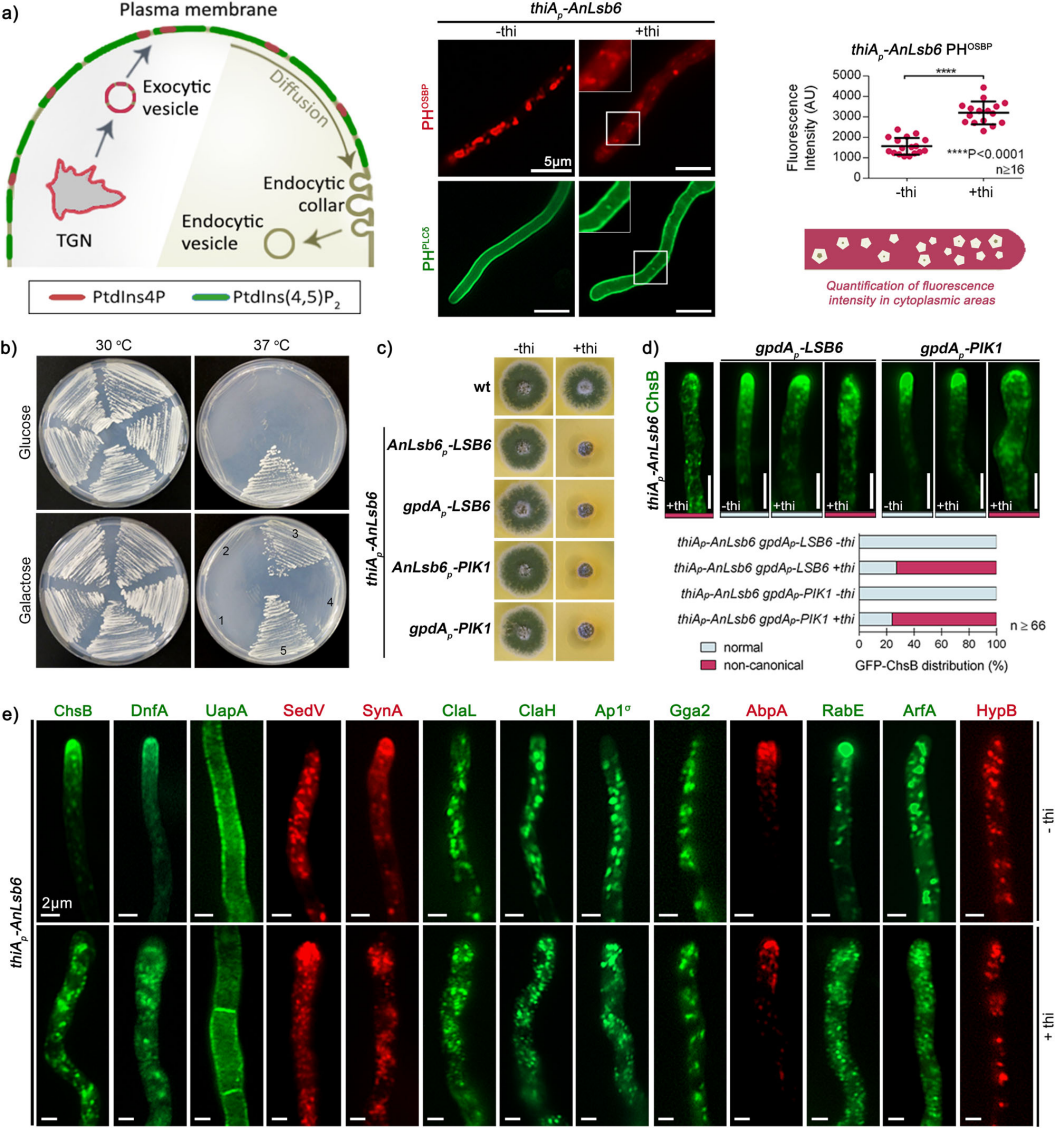
AnLsb6 affects the action of PtdIns4-specific sensors and heterologously complements thermosensitivity in a *PIK1Δ* mutant. **a** Cellular distribution of PtdIns4P and PtdIns(4,5)P_2_ as monitored by the fluorescence of the PtdIns4P- and PtdIns(4,5)P_2_-specific sensors, the pleckstrin homology domains of the oxysterol binding protein (PH^OSBP^) and the rat phospholipase C1 (PH^PLCδ^), in conditions of AnLsb6 depletion ( + thi). Whereas PH^PLCδ^ localization remains largely undisturbed at the plasma membrane with the exception of a few scattered spots, an almost complete disorganization of the polarized structures recognized by PH^OSBP^ and an increase of cytoplasmic haze (2.04-fold; *P* < 0.0001; -thi *n* = 17, +thi *n* = 16) is observed in the case of PH^OSBP^. Raw data on the quantification of fluorescent cytoplasmic areas are given in Supplementary Data 3. See Methods for statistical analysis and statistical tests used. Error bars represent SD. **b** Growth of *S. cerevisiae* PIK1Δ strains expressing the empty vectors pFL038 (Nr. 1) and pCG563 (Nr. 2), AnLsb6 driven under the yeast PIK1 promoter (Nr. 4) or overexpressed under the galactose responsive galactokinase promoter GAL1 (Nr. 3), compared to the wt (Nr. 5) in the presence of appropriate carbon sources (2% Glucose, 2% Galactose). Growth was assessed on minimal media, at 30^o^ and 37^o^ C for 96 h. **c** Growth test of LSB6 and PIK1 expressed under the native *Anlsb6* promoter or overexpressed under the glyceraldehyde-3-phosphate dehydrogenase promoter *gpdA*, at conditions where AnLsb6 is expressed (-thi) or repressed by thiamine ( + thi). Growth was assessed on minimal media, pH 6.8, 37^o^ C for 72 h. Fluorescence microscopy and quantification of ChsB rescue for the strains harboring LSB6 and PIK1 under the *gpdA* promoter is shown in (**d**). The wt GFP-tagged ChsB resembles the condition where AnLsb6 is expressed in the gpdA_p_-LSB6 and gpdA_p_-PIK1 strains (for a -thi GFP-ChsB control see Fig. 3e). Colors: Light gray, normal; Dark pink, non-canonical. For quantification of thiA_p_-AnLsb6 GFP-ChsB +thi, see also Supplementary Fig. 5. Raw data on the quantification of growth are given in Supplementary Data 5 (thiA_p_-AnLsb6 gpdA_p_-LSB6 -thi *n* = 172; thiA_p_-AnLsb6 gpdA_p_-LSB6 +thi *n* = 108; thiA_p_-AnLsb6 gpdA_p_-PIK1 -thi *n* = 66; thiA_p_-AnLsb6 gpdA_p_-PIK1 +thi *n* = 112). **e** Cellular localization of GFP- or mRFP/mCherry-tagged protein cargos under conditions where AnLsb6 is expressed (upper panel) or repressed by thi (lower panel, +thi). The cargoes tested are the chitin synthase ChsB, the flippase DnfA, the transporter UapA, the t-SNARE SedV^SED5^, the synaptobrevin homologue SynA, clathrin light and heavy chains ClaL and ClaH, the adaptors Ap1σ and Gga2, the actin related protein AbpA^ABP1^, the Rab GTPase RabE^RAB11^, the GTPase ArfA^ARF1^ and the GEF HypB^SEC7^. Notice the misplacement or disorganization of most proteins, with the exception of the plasma membrane transporter UapA that is largely unaffected.

The results on strong signal overlap of AnLsb6 with Golgi membranes and especially with the PH^OSBP^ fluorescent sensor^56^, strongly indicate that this phosphoinositide kinase is involved in the generation of PtdIns4P. Given the fast interchangeable phosphorylation status of the PtdIns head group, difficulties in proving specific PtdIns phosphorylation and the ongoing efforts to generate appropriate tools to study PtdIns^2,57–59^, the established fluorescence sensors carrying the pleckstrin homology domains of the human oxysterol binding protein (PH^OSBP^) and of the rat phospholipase C1 (PH^PLCδ^)^45^ were used to monitor the effects of AnLsb6 depletion at the levels of PtdIns4P and PtdIns(4,5)P_2_ enriched membranes, respectively. Figure 3a shows that *AnLsb6* repression leads to massive disorganization of the characteristic polarized cisternae-like structures recognized by PH^OSBP^ and also to an increase of cytoplasmic haze (2.04-fold increase, *P* < 0.0001; *n* ≥ 16; Supplementary Data 3). Nevertheless, several scattered foci were still detectable, however these may well reflect areas of mis-organized ArfA, since PH^OSBP^, at least in *S. cerevisiae*, is recruited to the Golgi through the combinatorial recognition of PtdIns4P and Arf1^56^ (Supplementary Fig. 4 and Supplementary Data 4). On the contrary, the characteristic bright plasma membrane associated fluorescence of PH^PLCδ^ remained in principal unaffected upon depletion of AnLsb6, with the exception of a limited number of isolated fluorescent spots throughout the hyphae. These observations suggest that AnLsb6 is influencing the abundance and maintenance of PtdIns4P-containing membranes primarily at the level of Golgi and possibly also affecting overall PtdIns4P levels at the plasma membrane.

Given the above observations, it becomes exciting to ask whether this phosphoinositide kinase also incorporates the functions of the missing Pik1p homologue. The Golgi-resident Pik1p is the main contributor of PtdIns4P in *S. cerevisiae* and is recruited by Arf1 in order to facilitate and regulate proper vesicle formation, among other functions^8,18^. These essential cellular reactions are reflected by the strict thermosensitive phenotype of *PIK1Δ* strains^17^. This phenotype was utilized to check if AnLsb6 is able to restore at least some of the essential functions of Pik1p in *S. cerevisiae*. Figure 3b shows that indeed a heterologous expression of the *A. nidulans AnLsb6* cDNA overexpressed under the galactose responsive galactokinase promoter *GAL1*, and not under the native PIK1 promoter, is able to rescue a Pik1p deletion strain from thermosensitivity. On the contrary, heterologous expression of *S. cerevisiae LSB6* and *PIK1* cDNAs under the native *AnLsb6* promoter or overexpressed under the strong glyceraldehyde-3-phosphate dehydrogenase promoter *gpdA*, was not able to compensate for *AnLsb6* repression at the growth test level, nevertheless resulting, to a minor extent, in partial re-routing towards the plasma membrane (Fig. 3c, d and Supplementary Data 5).

### AnLsb6 repression affects the subcellular localization of polar cargoes

The effect of AnLsb6 depletion in the distribution of the PH^OSBP^ sensor and consequently in the cellular PtdIns4P levels should also be reflected in the localization of other proteins following the canonical PtdIns4P-dependent secretion route. Previous work in fungi has shown that Golgi derived vesicles may be delivered both to the plasma membrane and the endosomal system by involving a concerted action of PtdIns4P, adaptors and coats, as well as specific GTPases and that these routes may be interconnected^8,54,60–67^. In this context, several available cellular markers and cargoes labeling from early Golgi compartments up to the plasma membrane and the hyphal apex were monitored under *AnLsb6* induced and repressed conditions. These include the chitin synthase ChsB^68,69^, the phospholipid flippase DnfA^70^, the early Golgi t-SNARE SedV^SED5^ ^53^, the plasma membrane uric acid/xanthine transporter UapA^71^, the synaptobrevin homologue SynA^72^, clathrin light and heavy chains ClaL and ClaH^51,52^, the post Golgi associated clathrin adaptor complex domain Ap1σ^54^, the homologue of the trans Golgi-resident adaptor Gga2p^60,73^, the AbpA^ABP1^ actin binding protein^72^, the secretory RabE^RAB11^ GTPase^61,64^, the ArfA^ARF1^ Ras GTPase^47,48^ and the HypB^SEC7^ Arf1 GEF^45,49^. Representative images in Fig. 3e show that most tested proteins appear rather disorganized and/or scattered except for UapA that remains unaffected, SedV^SED5^ that appears to be accumulating close to the apex, as well as HypB and Gga2 that appear more misplaced towards the hyphal apex. Particularly interesting is the disorganization of RabE^RAB11^ and of SynA, which are involved in vesicle exocytosis^61,65,72^, indicating problematic secretion and recycling. It should also be noted that the localization of the various cellular markers was not always homogeneously affected and in certain cases, as highlighted for ChsB and DnfA (Supplementary Fig. 5 and Supplementary Data 6), cargo manages to reach their expected destinations in morphologically distorted hyphae. Nevertheless, the appearance of these phenotypes never exceeded 18.2% of the tested hyphal cells and in most cases the misplaced cargo appeared sorted as a more or less patchy network towards the hyphal apex^74–76^. This network may reflect accumulations of stranded late-Golgi associated membranes^77^, as is also indicated by the higher degrees of colocalization of different late and post-Golgi markers upon repression of *AnLsb6* with the exception of HypB^SEC7^ (Supplementary Fig. 6 and Supplementary Data 7).

### AnLsb6 repression leads cargo to RabA/B^RAB5^-dependent arrest at endosomal compartments

**Fig. 4:**
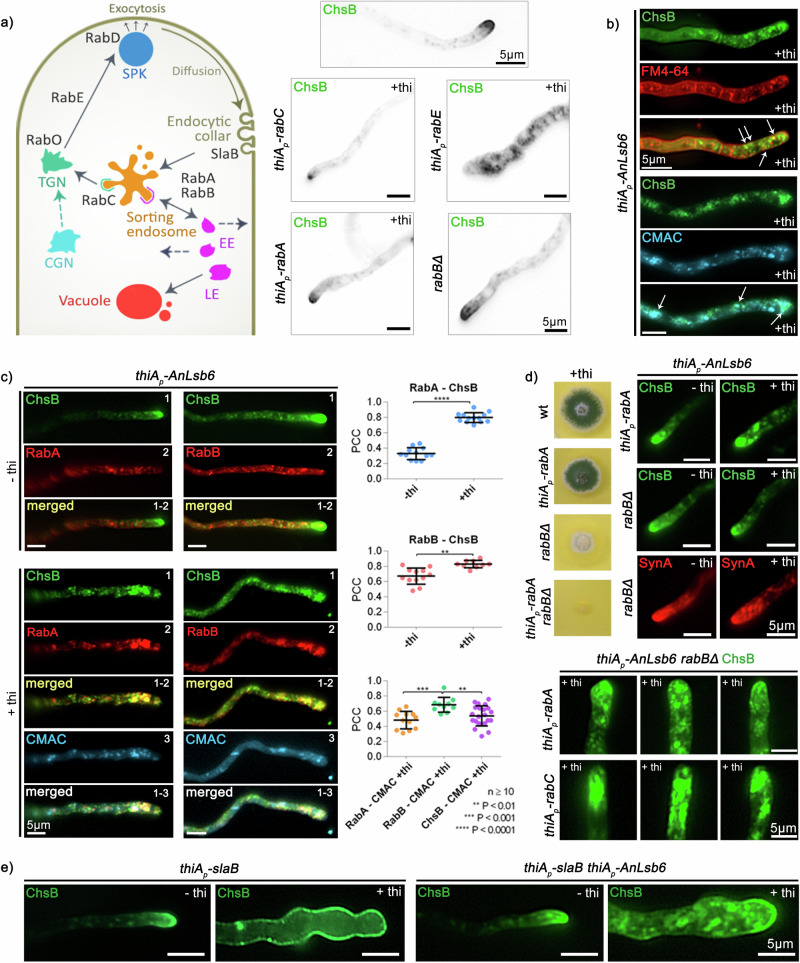
AnLsb6 repression leads to RabA/B^RAB5^-dependent endosomal arrest of the chitin synthase ChsB. **a** Speculative scheme of components of the endocytic recycling pathway at the hyphal tip, adopted and modified from Hernández-González et al.^77^. Subcellular localization of ChsB in strains carrying single *thiA-rabC*, *thiA-rabE*, and *thiA-rabA* at repressive conditions in the presence of thiamine ( + thi), as well as in a genetic background where *rabB* is deleted. **b** Localization of ChsB in the presence of FM4-64 and of the vacuolar stain CMAC. **c** Colocalization and relative quantification of GFP-ChsB with mRFP-tagged versions of RabA and RabB under conditions where AnLsb6 is expressed (-thi) or repressed by thiamine ( + thi) (RabA-ChsB: -thi PCC = 0.33 *n* = 12, +thi PCC = 0.80 *n* = 13, *P* < 0.0001; RabB-ChsB: -thi PCC = 0.67 *n* = 12, +thi PCC = 0.83 *n* = 10, *P* = 0.0093; RabA-CMAC PCC_A_ = 0.48 *n* = 12, RabB-CMAC PCC_B_ = 0.68 *n* = 10, ChsB-CMAC PCC_C_ = 0.54 *n* = 22, PCC_A_-PCC_B_ *P* = 0.0002, PCC_B_-PCC_C_ P = 0.0050). Raw data on the quantification of co-localization by calculating Pearson’s Correlation Coefficient (PCC) are given in Supplementary Data 8. Error bars represent SD. See Methods for statistical analysis and statistical tests used. **d** Growth of single and double mutants of *thiA-rabA*, *rabBΔ* and subcellular localization of apical cargos (ChsB, SynA) in *rabBΔ* or *thiA-rabA* genetic backgrounds and at conditions where AnLsb6 is expressed (-thi) or repressed by thiamine ( + thi). Localization of ChsB in a *rabBΔ* background and at conditions where both AnLsb6, RabA and RabC are repressed by thiamine ( + thi). **e** Subcellular localization of ChsB in a strain carrying a single *thiA-slaB* allele in the absence or presence of thiamine, and at conditions where both AnLsb6 and SlaB^SLA2^ are either expressed (-thi) or repressed by thiamine ( + thi).

The above results indicate that cargoes following the so-called “canonical” secretory pathway are misled, stranded or blocked at a specific secretory stage upon repression of AnLsb6 levels. In an attempt to gain further insight into the nature of this vesicular network, we made further use of the chitin synthase ChsB, an essential protein, extensively used as a model cargo to study the apical secretory routes^54,68,69,76–79^, the v-SNARE synaptobrevin homologue SynA involved in secretion and following the same polarizing routes via indirect endocytic recycling as ChsB^72,77,80^, as well as of the accumulated knowledge on *A. nidulans* Rab-GTPases as key regulators of intracellular trafficking^65^. ChsB follows canonical secretion via polarized exocytosis through the action of RabE^61^, involving the insertion to the plasma membrane at the apex, followed by diffusion towards the subapical endocytic collar, where it enters endocytic vesicles leading to a sorting endosome, operating upstream of RabB^RAB5^ enriched membranes. By engaging dynein complexes, ChsB-containing vesicles then either follow the RabB^RAB5^-dependent endocytic pathway or recycle to the Golgi in a RabC^RAB6^-dependent manner and adopt the route to the apex under the regulation of RabE^RAB11^, possibly also assisted by other Rab GTPases^53,61,65,77^. Figure 4a shows that with exception of RabE^RAB11^ and to a lesser extent RabC, the depletion of other Rab-GTPases involved in the intracellular trafficking pathways of ChsB is not reflected in a mis-localization of this cargo, suggesting the existence of alternative routes. In this context, and given also the slight signal overlap between ChsB and FM4-64 or CMAC in conditions of AnLsb6 depletion (Fig. 4b), we checked for potential colocalization of the misplaced/stranded ChsB-containing vesicles with Rab5-decorated membranes. *A. nidulans* has two Rab5 paralogues, RabA and RabB, that are essential as double mutants and that have relatively distinct topologies, the RabA being also more motile. Both are able to recruit the Corvet-complex, the RabB however being the main contributor in the processes of endosome maturation to vacuoles by recruiting Vps34, the PtdIns3P-synthesizing kinase required for initiating the multivesicular body pathway^80,81^. Figure 4c shows that upon AnLsb6 repression, ChsB exhibited a relatively high degree of overlap with both RabA and RabB, and especially with RabA at regions closer to the hyphal apex (Supplementary Data 8). This observation prompted us to investigate whether RabA and RabB also have a more active role in these processes. Surprisingly in this context, an inactivation of RabB and to a much lesser extent of RabA, resulted in release of accumulated vesicles towards the hyphal tip (*n* ≥ 40; 84.6% and 46.3% of hyphal tips, respectively), suggesting functional restoring of the relevant recycling machinery and that RabB is either promoting or preventing the recruitment of effectors responsible for further trafficking (Fig. 4d). The colony growth defect caused by the depletion of AnLsb6 is nevertheless maintained in a *rabB*Δ strain (not shown), and SynA recycling is also not entirely (*n* = 11; 63.6%) restored (Fig. 4d), indicating RabB-independent essential functions of AnLsb6. More surprisingly, this RabB-dependent apparent release is again blocked when both RAB5 paralogues are inactivated (*n* ≥ 32; 89.2% of hyphal tips), leading to accumulation of trapped vesicles underneath the apical dome, at a patch resembling the accumulation in dynein mutants^77,80,82^. This suggests that when AnLsb6 is depleted, vesicles may bypass the central secretory hub regulated by RabE^RAB11^ at the TGN-post Golgi interface and reach the plasma membrane less efficiently through the early endosome-mediated peripheral recycling route, as also shown for *S. cerevisiae*^83^. Moreover, when AnLsb6 and the two Rab5 paralogues are depleted, vesicles are trapped in the dynein/kinesin 1 loading zone, suggesting that the actin cables proximal to the tip region cannot act independently in the exocytic process^69,79,80,82^. The trafficking trajectories are even further highlighted in strains depleted for AnLsb6, RabB and RabC, where the appearance of accumulated vesicles is more prominent (*n* ≥ 32; 62.5% of hyphal tips) (Fig. 4d). In a similar context, ChsB was monitored under depleting conditions for both AnLsb6 and SlaB^SLA2^. The latter is a regulator that controls endocytosis at the collar region, the mutations of which result in polarity maintenance failure and distribution of cargo throughout the plasma membrane, rather than polarized at the apical dome^52,54,77^. Figure 4e shows that although the effects of *slaB* repression prevailed, resulting in abnormal cells with homogeneous distribution of ChsB at the plasma membrane, accumulated vesicles and clumps still can be detected intracellularly towards the germling apex in AnLsb6 repressed conditions, underscoring the implication of AnLsb6 in the secretory trafficking routes of *A. nidulans*.

## Discussion

Studies on Lsb6p and its homologues in other fungi have assigned auxiliary roles in the decoration of PtdIns4P on relevant membranes. In *S. cerevisiae*, Lsb6p has been identified as a non-essential plasma- and vacuolar membrane associated type II phosphatidylinositol 4-kinase, involved in PtdIns4P generation and actin-dependent endosome motility^28,29^, that also binds Las17p, a homolog of the human Wiskott-Aldrich Syndrome protein, involved in actin patch assembly and actin polymerization^25,26,28,29^. An association with PtdIns4P generation on endosomal membranes has also been proposed for the non-essential homologous protein FgLsb6 in *F. graminearum*^36^. Concerning the localization of the other PtdIns4P kinases in *S. cerevisiae*, Stt4p is localized in the plasma membrane^14^ and Pik1p localizes to the Golgi, but is also capable to translocate to the nucleus^17,19^. This, along with the facts that PtdIns4P kinases Pik1p and Stt4p do not seem to possess overlapping roles as overexpression of either gene does not suppress the essentiality of the other, that Lsb6p can also partially suppress the lethal phenotype of a mutant lacking functional Stt4p but not of Pik1p^26^, that AnLsb6 is associated with Golgi membranes and its size is larger than that of Lsb6p (775 vs 607 aa), as well as the observation that Eurotiomycetes sequenced so far appear to lack homologues of Pik1p, makes the assumption that AnLsb6 and Pik1p have at least some specific overlapping functions an intriguing hypothesis. In this study, we provide evidence that AnLsb6, apart from associating with vesicular structures overlapping with established markers of the Golgi, when depleted, it also leads to a pronounced disorganization of PtdIns4P-decorated membranes recognized by the PtdIns4P-specific sensor PH^OSBP^. These correlations, plus the fact that heterologous overexpression of AnLsb6 is able to compensate for the thermosensitivity phenotype of a Pik1p deletion mutant in *S. cerevisiae* and vice versa, albeit to a minor extent, suggests that AnLsb6 may have adopted at least some of the Pik1p proposed functions, like for example the supply of PtdIns4P-containg scaffolds for recruitment of effectors or other associated proteins involved in vesicle formation and trafficking^1^. This may be reflected by the demonstrated close proximity of AnLsb6 with GEF HypB^SEC7^ shown by bimolecular fluorescence (BiFC), and the influence of depleted HypB^SEC7^ and clathrin heavy chain ClaH on AnLsb6 localization or vice versa. These results may also correlate with the analogous physical interaction of Pik1p with Sec7p in *S. cerevisiae*, proposed to operate as a dual signal for specific recruitment of clathrin coats to the late Golgi, along with Arf1p^84^, a result challenged however by others proposing that Arf1p is the actual partner of Pik1p^18^. In this context it would be most interesting to also know if AnLsb6 is able to interact with ArfA^ARF1^ or even the other established early Golgi marker, Arf1 GEF GeaA^GEA1^ ^50^. In an attempt to further illustrate the significance of AnLsb6 in PtdIns4P generation, the localization of intracellular markers associating with these trafficking processes was monitored. Most markers of the secretory pathway appeared misplaced and/or disorganized at different degrees, with the HypB and the Gga2p-homologue Gga2 being much less affected, suggesting that the consequence of the AnLsb6 action is shifted towards the late- and post-Golgi levels. Interestingly, the localization of the clathrin adaptors Ap1 and Gga2, the recruitment of which is being regulated by the cellular PtdIns4P levels in *S. cerevisiae*, are responding differently to the AnLsb6 depletion in *A. nidulans*^8,60,63^. Concerning cargoes, apart from the transporter UapA, that was also previously shown to be able to bypass the Golgi dependence on the way to the plasma membrane^48,55,85^, the chitin synthase ChsB and the flippase DnfA, as passengers of the endocyting recycling pathway are unable to efficiently reach the hyphal apex and appear either as scattered foci, or accumulated as fluorescent spots underneath the apical dome, suggesting a defective polarization through indirect endocytic recycling^51,77^. The latter is also highlighted by the defect in the localization of the synaptobrevin homologue SynA, a well-established marker of this recycling pathway^77,80^. Interestingly, the actin binding protein AbpA^ABP1^, a marker of endocytic patches, also appears to be affected to some extent, indicating an influence on the function of the hyphal collar, which might have further implications on the actin cytoskeleton organization, as implied by the actin-dependent endosome motility on Lsb6p. As such, this result speculatively provides a link towards interactions with the less characterized Las17p and the Arp2,3-complex homologues in *A. nidulans*, further suggesting that AnLsb6 also maintains characteristics of Lsb6p, besides adopting Pik1p functions^28,29,86–89^.

So, could these accumulations of vesicles at AnLsb6-limiting conditions be reflecting a form of the otherwise “enigmatic” sorting endosome^90^, accommodating heterogeneous early and late endosomes from the anterograde and retrograde recycling pathways and are PtdIns4Ps indispensable for all these trafficking routes? Studies on ChsB targeting proposed an upstream compartment, as a sort of central hub accommodating features of both anterograde and retrograde trafficking routes^77^. Colocalization of ChsB-containing vesicles with both Rab5 homologues and especially with their more motile part consisting of RabA^80,91^, suggests the predominant presence of early endosomes and to a lesser extent CMAC-stained mature endosomes, at conditions of AnLsb6 depletion. Concerning the simultaneous limitation of both AnLsb6 action and endocytosis at the hyphal collar region, results suggest that incoming vesicles from the collar during endocytosis may be sorted in this compartment. RabA-dependence of ChsB targeting in the absence of RabB upon ALsb6 depletion, nevertheless, makes an AnLsb6-dependent direct sorting route from endosomes to the plasma membrane a plausible possibility, unless RabA-positive endosomes are rerouted towards the Golgi and travel in anterograde direction dependent on RabE^Rab11^ or other Rab GTPases, but independent of PtdIns4P. These possible routes are also reflected in the percentage of hyphae appearing as wt (37.5%) in strains where AnLsb6, RabB and RabC are depleted. Mis-sorting due to PtdIns4P-dependent failure in the recruitment of other crucial effectors, or in their timely interaction, or their indirect involvement, is of course also possible, for example a problematic attachment, or action of other endosomal specific PtdIns-kinases and phosphatases (e.g. Vps34, Sac1) generating distinct PI species that cannot be further processed^2,3^, or a defect in the coordination of specific GTPases and other accessory proteins required for secretion and membrane remodeling^78,92^, like the Arf1, the Arf1 GEF HypB^SEC7^, or the RabC^RAB6^ / GARP tethering complex^77,93,94^, SarA^SAR1^^95^ or the AP-1 complex and clathrin adaptors^5,54,65,77,88,96,97^. Cytoskeleton components and accessory proteins like the dynein/kinesin 1 or the type V myosin MyoE^64,69,82,98–100^, or components of the CORVET and the retromer complexes^101–104^ may also be influenced. In any case, the fact that AnLsb6 and also the endosome to Golgi route regulator RabC^53,77^ are not essential for survival, implies that an arrest at a limiting intermediate vesicular compartment is only transient, or represents a massive stalling in the absence of these proteins, or even that this route can also be bypassed, suggesting that AnLsb6 is not the only kinase affecting PtdIns4P enrichment, most probably being assisted, or partially replaced by the other essential PtdIns4P-kinase AnStt4^32^. Nevertheless, the type II PtdIns4-kinase AnLsb6 appears to be the essential determinant for navigating cargo following the endocytic recycling trafficking pathway through an intermediate compartment, presumably with sorting endosome identity, and besides its general impact in the mechanisms governing secretion, it may provide a suitable target in combating fungal pathogens within the Eurotiomycetes, like *A. fumigatus*. More studies are necessary on PtdInsP and relevant kinases/phosphatases in order to unravel specific details in their role serving as docking stations for recognition by protein partners regulating complex membrane dynamics.

## Methods

### Media, strains and transformation

Standard complete and minimal media for *A. nidulans* as well as medium supplements were used (details in FGSC; http://www.fgsc.net/Aspergillus/gene_list/media.html and http://www.fgsc.net/Aspergillus/gene_list/supplement.html) Chemicals and reagents were obtained from Sigma-Aldrich (Life Science Chemilab SA, Hellas) or AppliChem (Bioline Scientific SA, Hellas). For *A. nidulans* minimal media, carbon sources were used at concentrations of 0.1-1% (w/v) and nitrogen sources, sodium nitrate (NaNO_3_) and ammonium tartrate [(NH_4_)_2_C_4_H_4_O_6_], were used at final concentrations of 10 mM. For *S. cerevisiae* minimal media (BD Difco Yeast Nitrogen Base), carbon sources were used at concentrations of 2-3% (w/v) and ammonium sulfate [(NH_4_)_2_SO_4_] at a final concentration of 20 mM was used as a nitrogen source. Thiamine hydrochloride (thi) was used at a final concentration of 10 μM and in all cases it was applied to the cultures ab initio. Lithium acetate-based transformation in *S. cerevisiae* was performed as described in ref. ^105^. Transformation in *A. nidulans* was performed as described in ref. ^106^ using a *nkuA* DNA helicase deficient recipient strain^107^. Except for the strains expressing *S. cerevisiae* LSB6 and PIK1 cDNAs, all other *A. nidulans* strains carrying tagged versions and promoter switches are in-locus gene replacements. Targeted integrations of gene fusions were achieved by amplification of linear cassettes also carrying the markers orotidine-5’-phosphate-decarboxylase (*AFpyrG*, Afu2g0836), GTP-cyclohydrolase II (*AFriboB*, Afu1g13300) and a pyridoxine biosynthesis gene (*AFpyroA*, Afu5g08090) from *A. fumigatus*, resulting in complementation of the relevant auxotrophies. Combinations of tagged strains were generated by standard genetic crossing. Plasmid expression in *S. cerevisiae* was selected by complementation of the relevant auxotrophic mutations URA2, LEU2, HIS3, and MET25. Transformants were verified by PCR. *E. coli* DΗ5α strain was used for cloning purposes. Strains used in this study are listed in Supplementary Table 1.

### Nucleic acid manipulations and plasmid constructions

Genomic DNA extraction was performed as described in FGSC. RNA extraction was performed using the TrizolTM reagent according to the manufacturer’s instructions (Thermo Fischer Scientific). Conventional and high-fidelity PCR reactions were performed using KAPA Taq DNA and Kapa HiFi polymerases (Kapa Biosystems, Roche Diagnostics). cDNA synthesis and RT-PCR were performed using the QuantiTect Reverse Transcription Kit according to the manufacturer’s instructions (Qiagen, SafeBlood Bioanalytica SA, Hellas). Nucleospin Plasmid kit and the Nucleospin Extract II kit (Macherey-Nagel, Lab Supplies Scientific SA, Hellas) were used for plasmid preparation and DNA gel extraction. Restriction enzymes were from Takara Bio (Lab Supplies Scientific SA, Hellas). DNA sequencing was performed by Eurofins-Genomics (Vienna, Austria). Genomic DNA from the strain TNO2A7 was used as a template to amplify relevant fragments. Plasmids PDV7 and PDV8 were used as templates to amplify N- and C-terminal halves of YFP for bimolecular fluorescence^108^. Plasmids p1439 and p1491 were used as templates to amplify the Gly-Ala (5GA) linker fused with sGFP or mRFP, respectively^109^. Plasmids pFL038, pCJ313 and pCJ563 were used to complement auxotrophies and to introduce relevant cDNAs and *S. cerevisiae* gene fusions^105^. A pGEM-T based plasmid carrying an attenuated version of the gpdA promoter ( ~ 1000 bp) was used to heterologously overexpress relevant *S. cerevisiae* cDNAs complementing for the D-pantothenic acid auxotrophy^110^. In this plasmid the corresponding sequence of the *gpdA* promoter sequence was replaced by ~1000 bp of the putative AnLsb6 promoter. Gene fusions in *A. nidulans* were generated by one step ligations or sequential cloning of the relevant fragments in the plasmids pBluescript SKII, or pGEM-T using specific primers carrying additional restriction sites, or by fusion PCR^109^. The resulting plasmids were used as templates for linear cassette amplification by PCR. Gene fusions in *S. cerevisiae* were generated in vivo by transforming the relevant linear fragments in appropriate *S. cerevisiae* strains. Oligonucleotides used for these constructs are found in Supplementary Table 2.

### Protein extraction and western blot

Cultures for total protein extraction were grown in minimal media (pH 6.8) supplemented with NH_4_^+^ at 25 ^o^C for 18 h at 140 rpm. Total protein extraction was performed from mycelia using a fungal protease inhibitor cocktail (Sigma‐Aldrich, Life Science Chemilab SA), as previously described in ref. ^44^. Total proteins (25–50 μg, estimated by Bradford assays) were separated in 8–10% w/v polyacrylamide gels (Mini Protean^TM^ Tetra Cell, Bio-Rad) and were electroblotted on nitrocellulose membranes (Amersham Protran^TM^ Premium) using the Bio-Rad Trans-Blot® Turbo^TM^ transfer system. Immunodetection was performed with a primary anti GFP D5.1 (Cell Signaling Technology, #2956), a primary AbFlex® Histone H3 antibody (Active Motif, #91300) and a secondary anti-rabbit IgG-HRP (Cell Signaling Technology, #7074). Images were acquired and documented using a Bio-Rad XRS+ ChemiDoc^TM^.

### Microscopy and quantification

Samples for epifluorescence microscopy were prepared as previously described in ref. ^52^. Germlings were incubated in 35 mm μ-dishes, high glass bottom (ibidi GmbH) in liquid minimal media for 18–20 h at 25 °C. Staining with FM4‐64 and Blue CMAC (7‐amino‐4‐chloromethyl coumarin) (Life Technologies, Molecular Probes) was according to Peñalva^43^ and Evangelinos et al.^44^, respectively. Brefeldin A was used at a final concentration of 100 μg ml^-1^. Images were obtained using an inverted Zeiss Axio Observer Z1 with appropriate filters and a Hamamatsu Orca Flash 4.0 LT Plus camera equipped with a W-View Gemini image splitter for simultaneous dual wavelength imaging. Contrast adjustment, area selection and color combining were achieved using the Zeiss-Zen 2012 software. Images were further processed and annotated in Adobe Photoshop CS4 Extended version 11.0.2. For fluorescence intensity measurements in a selected region of interest, the Area Selection tool of ICY software was used (http://icy.bioimageanalysis.org/), and for intensity profile plotting, the ImageJ Plot profile was used (https://imagej.nih.gov/ij/). For quantifying colocalization, Pearson’s Correlation Coefficient (PCC) above thresholds for a selected Region of interest (ROI) was calculated using the ICY colocalization studio plugin (pixel-based method) for single planes. In Fig. 2 the ROIs included the foci and the tip areas and were selected using the Spot Detector plugin, and/or the Area Selection tool. In all cases the fluorescence of the background was subtracted. In Fig. 3a the ROIs included only the cytoplasmic areas and were selected using the Area Selection tool. In Fig. 4c and Supplementary Fig. 6 the ROIs included only the subapical areas for the -thi condition, due to the high GFP signal intensities in the apical areas.

### Statistics and Reproducibility

Sample size corresponds to the total number of different hyphae of fungal strains of specific genotypes, observed and/or photographed. No data were excluded from the reported analyses. All microscopy experiments have been repeated independently as two to four biological repeats which correspond to different samples of fungal strains of specific genotypes, and more than eight technical replicates which correspond to different hyphae observed and/or photographed within each sample. The number of total replicates (biological and technical) for each fungal strain of specific genotype and in each culture condition is 8 to 111. All attempts at replication were successful. The significance of differences in PCCs was tested by one sample t-test. To test the significance in Fig. 3a, a two-tailed unpaired t-test was performed. Tukey’s Multiple Comparison test was performed (One-way ANOVA) for statistical analyses of three or more datasets. In all cases, confidence interval was set to 95% and analyses were performed using the GraphPad Prism software version 6.01.

### Reporting summary

Further information on research design is available in the Nature Portfolio Reporting Summary linked to this article.

### Supplementary information

Supplementary Information

Description of Additional Supplementary Files

Supplementary Data 1

Supplementary Data 2

Supplementary Data 3

Supplementary Data 4

Supplementary Data 5

Supplementary Data 6

Supplementary Data 7

Supplementary Data 8

Reporting Summary

## Data Availability

Source data for microscopy, fungal strains and other related materials are available upon reasonable request. Source data underlying the graphs in this study can be found in the Supplementary Data files and the uncropped/unedited western blot is shown in Supplementary Fig. 2.
