# Supplementary material for: A type II phosphatidylinositol-4-kinase coordinates sorting of cargo polarizing by endocytic recycling
Source: Commun Biol. 2024 Jul 12;7:855. doi: 10.1038/s42003-024-06553-3 (PMC11245547; doi:10.1038/s42003-024-06553-3)
Supplement: Supplementary file 1 — Supplementary Information [file 42003_2024_6553_MOESM1_ESM.pdf]

A type II phosphatidylinositol-4-kinase coordinates sorting of cargo polarizing by endocytic recycling

Anezia Kourkoulou[1], Olga Martzoukou[1], Reinhard Fischer[2] and Sotiris Amillis[1,2,#]

1. National and Kapodistrian University of Athens, Department of Biology, Athens, Hellas, Greece.
2. Karlsruhe Institute of Technology - South Campus, Institute for Applied Biosciences, Department of Microbiology, Karlsruhe, Germany.

[#] Correspondence: samillis@biol.uoa.gr

Supplementary Material

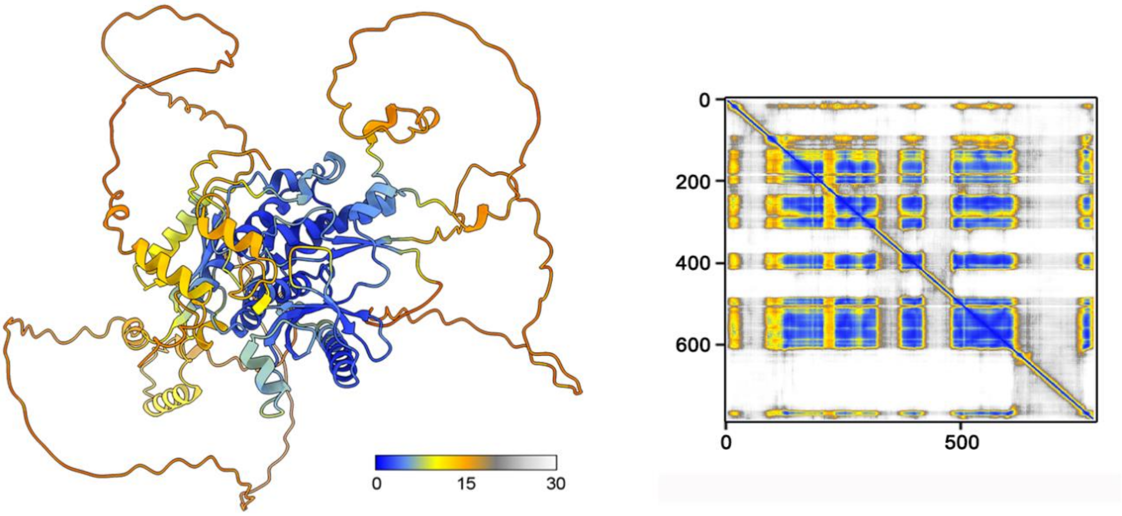

Supplementary Figure 1

Model of AnLsb6 generated with AlphaFold2 (left panel), colored based on Predicted Aligned Error (PAE). The plot of pairwise PAE values per residue is shown on the right panel. Notice the very high model confidence in the area predicted as the PI3/4 kinase domain (see also Figure 1a).

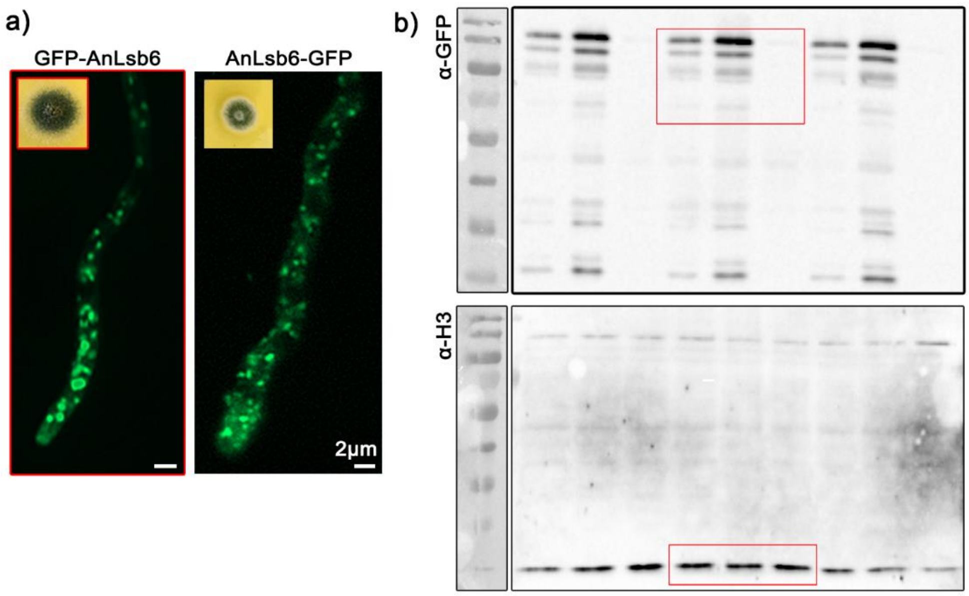

Supplementary Figure 2

a Comparison of N- and C-terminal GFP-tagged versions of ANLsb6. The image of GFP-AnLsb6 and the embedded growth test (highlighted with a red stroke) were also used in Figure 1B and 1C. **b** Uncropped images of the western blots. The cropped areas shown in Figure 1B are highlighted with a red stroke. The protein ladder PageRuler^{TM} Plus (Thermo Fischer Scientific) is also shown for orientation.

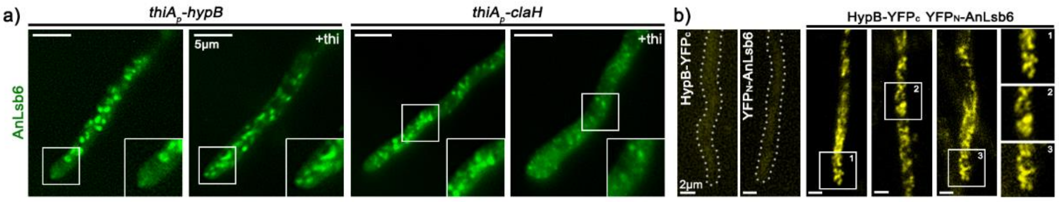

Supplementary Figure 3: AnLsb6 associates with other essential post-Golgi partners.

a Distribution of AnLsb6 at conditions of HypB^{SEC7} and ClaH repression (+thi) in isogenic strains carrying in locus thiamine-repressible alleles of *thiAp-hypB* and *thiAp-claH* compared to wt conditions (-thi). Notice the shift of AnLsb6 towards the hyphal apex under *hypB*-repressed conditions and the dis-organization of fluorescent polarized structures to scattered spots and cytoplasmic haze when clathrin ClaH is depleted, suggesting that a significant part of AnLsb6 is operating downstream of ClaH. **b** Bimolecular fluorescence (BiFC) analysis in strains carrying AnLsb6 and HypB^{SEC7} tagged with N-terminal YFP$_N$ and YFP$_C$, driven under the thiamine promoter *thiA* and the native *hypB* promoter, respectively. Fluorescence signals from single strains expressing either the YFP$_N$-AnLsb6, or HypB-YFP$_C$ are shown as controls.

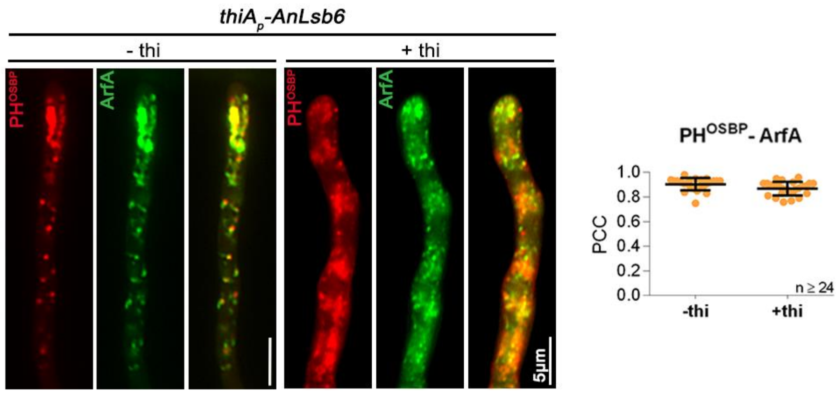

Supplementary Figure 4

Colocalization of PH^{OSBP} with ArfA^{ARF1} at conditions where AnLsb6 is expressed (-thi) or repressed by thiamine (+thi) (left panel), and quantification of PCC (right panel; PCC -thi = 0.91±0.05, P<0.0001, n=24; PCC +thi = 0.87±0.05, P<0.0001, n=27). Raw data on the quantification of co-localization by calculating Pearson's Correlation Coefficient (PCC) are given in Supplementary Data 4. Error bars represent SD.

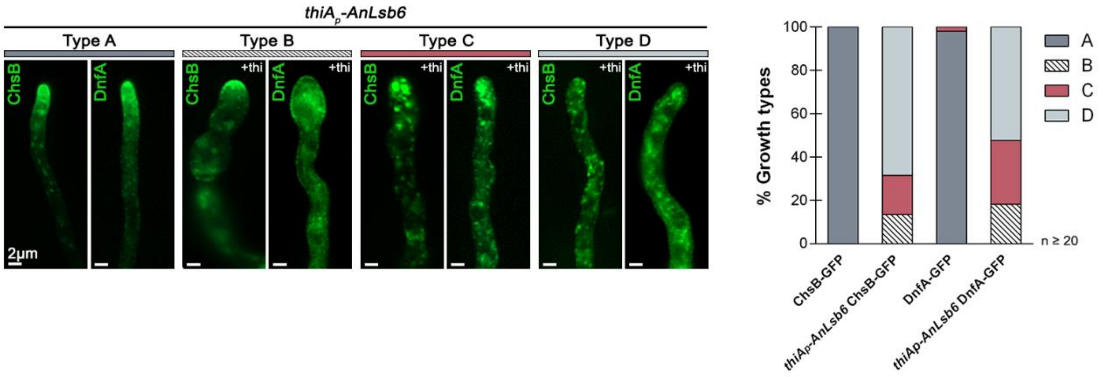

Supplementary Figure 5

Types of hyphal morphology and subcellular localization of the chitin synthase ChsB and the phospholipid flippase DnfA, observed and quantified under conditions where AnLsb6 is expressed or repressed by thiamine (+thi) (Type A, Dark gray; Type B, Diagonal line pattern; Type C, Dark pink; Type D: Light gray). Raw data on the quantification of growth are given in Supplementary Data 6 (ChsB-GFP, n=20; thiA$_p$-AnLsb6-GFP n=111; DnfA-GFP n=42; thiA$_p$-AnLsb6-GFP n=88).

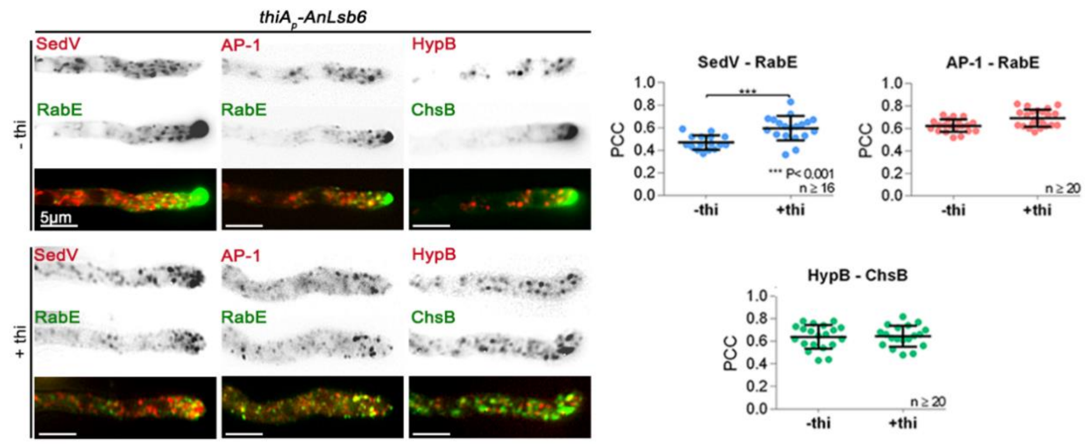

Supplementary Figure 6

Colocalization and relative quantification of GFP- or mRFP/mCherry-tagged protein cargos under conditions where AnLsb6 is expressed (-thi) or repressed by thiamine (+thi). Quantification of colocalization was performed in subapical areas only, for the condition -thi. Notice that, with the exception of the early Golgi marker SedV^{SED5} and the GEF HypB^{SEC7}, the fluorescence signal of all other proteins appears to overlap significantly at regions closer to the hyphal apex. Raw data on the quantification of co-localization by calculating Pearson's Correlation Coefficient (PCC) are given in Supplementary Data 7 (SedV-RabE: -thi n=16, +thi n=18; AP-1-RabE: -thi n=20, +thi n=23; HypB-ChsB: -thi n=20, +thi n=20). Error bars represent SD. See Methods for statistical analysis and statistical tests used.

Supplementary Table 1. Strains used in this study. All *A. nidulans* strains carry the *veA1* mutation affecting sporulation. *pabaA1, pyroA4, riboB2, argB2, pyrG89, pantoB100, biA1, nicA2* and *inoB2* are auxotrophic mutations for p-aminobenzoic acid, pyridoxine, riboflavin, arginine, uracil/uridine, D-pantothenic acid, nicotinic acid, biotin and inositol respectively. *yA2* and *wA4* are mutations resulting in yellow and white conidiospore colors respectively. *niiA4* is a loss of function mutant of the nitrite reductase. The *bar* gene encodes glufosinate resistance. *nkuA*Δ reduces the frequency of nonhomologous integration of transforming DNA fragments, improving gene targeting. 5xGA stands for a 5x Gly-Ala linker fused between ORF and the relevant tag. *ura3Δ0 leu2Δ0 his3Δ1 met15Δ0* are *S. cerevisiae* auxotrophic mutations for uracil, leucine, histidine and methionine, respectively.

**Strain**	**Genotype**	**Reference**
TNO2A7	*nkuAΔ::argB pyrG89 pyroA4 riboB2*	6
mRFP-PH^{OSBP}	*pyroA4[pyroA::gpdA$^{m}_{p}$::mRFP-PH^{OSBP}] inoB2 niiA4 wA4*	7
mCherry-sedV	*pyroA4[pyroA^{mut}::gpdA$^{m}_{p}$-mCherry-sedV] nkuAΔ::bar, wA4, niiA4 inoB2*	8
abpA-mRFP	*abpA-mRFP::AFpyrG yA2 pabaA1 pyrG89*	1
claL-GFP	*claL$^{-(5xGA)}$GFP::AFpyrG nkuAΔ::argB pyroA4 riboB2 pyrG89*	4
claH-GFP	*claH$^{-(5xGA)}$GFP::AFpyrG nkuAΔ::argB pyroA4 riboB2 pyrG89*	4
claH-mRFP	*claH$^{-(5xGA)}$mRFP::AFpyroA nkuAΔ::argB pyrG89 pyroA4 riboB2*	2
GFP-chsB	*GFP-chsB::AFpyrG nkuAΔ::argB pyroA4 riboB2 pantoB100*	2
dnfA-GFP	*dnfA-GFP::AFpyrG nkuAΔ::argB pyrG89 pabaA1 pyroA4*	10
mCherry-synA	*fwA1 pyrG89 pyroA4 nicA2 nkuAΔ::argB AFpyG-mCherry-synA yA::AFpyroA tpmAp-GFP-tpmA fwA1 nicA2*	11
GFP-$PH^{PLCδ}$	*[gpdA-GFP-$PH^{PLCδ}$]-argBmut::argB2 pabaA1 yA2*	7
uapA-GFP	*uapAΔ::uapA-GFP::AFriboB uapCΔ::AfpyrG nkuAΔ::argB pabaA1 pyroA4 riboB*	3
GFP-rabE	*GFP-RabE::AFpyrG nkuAΔ:argB pyrG89 pyroA4 riboB2*	4
ap1σ-GFP	*ap1σ$^{-(5xGA)}$GFP::AFpyrG nkuAΔ::argB pyrG89 pyroA4 riboB2*	5
thiA$_{p}$-hypB	*thiAp-hypB::AFpyrG nkuAΔ::argB pyrG89 pyroA4 riboB2*	2
thiA$_{p}$-claH	*thiAp-claH::AFpyroA nkuAΔ:argB pyrG89 pyroA4 riboB2*	4
thiA$_{p}$-rabA rabBΔ	*thiA$_{p}$-rabA::AFpyroA rabBΔ::AFpyrG nkuAΔ::argB pyrG89 pyroA4 riboB2*	2
arfA-GFP	*arfA-(5xGA)GFP::AFpyrG nkuAΔ::argB pyrG89 pyroA4 riboB2*	this study
hypB-mScarlet	*hypB$^{-(5xGA)}$mScarlet::AFriboB nkuAΔ::argB pyrG89 pyroA4 riboB2*	this study
gga2-GFP	*gga2$^{-(5xGA)}$GFP::AFpyrG nkuAΔ::argB pyrG89 pyroA4 riboB2*	this study
Anlsb6Δ	*Anlsb6Δ::AFpyrG nkuAΔ::argB pyrG89 pyroA4 riboB2*	this study
thiA$_{p}$-Anlsb6	*thiAp-Anlsb6::AFpyrG nkuAΔ::argB pyrG89 pyroA4 riboB2*	this study
thiA$_{p}$-FLAG-Anlsb6	*thiApFLAG-Anlsb6::AFpyrG nkuAΔ::argB pyrG89 pyroA4 riboB2*	this study
thiA-GFP-Anlsb6	*thiAp-GFP-Anlsb6::AFpyrG nkuAΔ::argB pyrG89 pyroA4 riboB2*	this study
GFP-Anlsb6	*GFP-Anlsb6::AFpyrG nkuAΔ::argB pyroA4 riboB2 pyrG89*	this study
Anlsb6-GFP	*Anlsb6$^{-(5xGA)}$GFP::AFpyrG nkuAΔ::argB pyroA4 riboB2 pyrG89*	this study
GFP-Anlsb6 mRFP-PH^{OSBP}	*GFP-Anlsb6::AFpyrG [pyroA::gpdAmp-mRFP-PH^{OSBP}]pyroA4 inoB2*	this study
GFP-Anlsb6 mCherry-sedV	*GFP-Anlsb6::AFpyrG pyroA4::[pyroA::gpdAmp-mCherry-sedV] nkuAΔ::argB inoB2*	this study
GFP-Anlsb6 ArfA-mRFP	*GFP-Anlsb6::AFriboB arfA$^{-(5xGA)}$mRFP::AFpyrG nkuAΔ::argB pyrG89 pyroA4 riboB2*	this study
GFP-Anlsb6 HypB-mScarlet	*GFP-Anlsb6::AFpyrG hypB$^{-(5xGA)}$mScarlet::AFriboB nkuAΔ::argB pyrG89 pyroA4 riboB2*	this study
GFP-Anlsb6 ClaH-mRFP	*GFP-Anlsb6::AFpyrG claH$^{-(5xGA)}$mRFP::AFpyroA nkuAΔ::argB pyrG89 pyroA4 riboB2*	this study
GFP-Anlsb6	*GFP-Anlsb6::AFriboB thiAp-hypB::AFpyrG nkuAΔ::argB pyrG89 riboB2 pyroA4*	this study

thiA$_p$-hypB		
GFP-Anlsb6<br>thiA$_p$-claH	*GFP-Anlsb6::AFpyrG thiAp-claH::AFpyroA nkuAΔ::argB pyrG89 riboB2 pyroA4*	this study
YFPn-AnLsb6	*thiAp-(5xGA)YFPn-*AnLsb6*::AFpyrG nkuAΔ::argB pyrG89 pyroA4 riboB2*	this study
hypB-YFPc	*hypB-(5xGA)YFPc::AFriboB nkuAΔ::argB pyrG89 pyroA4 riboB2*	this study
YFPn-AnLsb6<br>hypB-YFPc	*thiAp-(5xGA)YFPn-* AnLsb6*::AFpyrG hypB-(5xGA)YFPc::AFriboB nkuAΔ::argB pyrG89 pyroA4 riboB2*	this study
thiA$_p$-Anlsb6<br>mRFP-PH^{OSBP}	*thiAp-Anlsb6::AFpyrG pyroA4::[pyroA-gpdApm-mRFP-PH^{OSBP}] riboB2*	this study
thiA$_p$-Anlsb6<br>GFP-chsB	*thiAp-Anlsb6::AFpyrG GFP-chsB::AFpyrG nkuAΔ::argB pyroA4 riboB2 pantoB100*	this study
thiA$_p$-Anlsb6<br>dnfA-GFP	*thiAp-Anlsb6::AFpyrG dnfA-GFP::AFpyrG nkuAΔ::argB pyroA4*	this study
thiA$_p$-Anlsb6<br>mCherry-synA	*thiAp-Anlsb6::AFpyrG AFpyrG-mCherry-synA nkuAΔ::argB pyroA4*	this study
thiA$_p$-Anlsb6<br>uapA-GFP	*uapAΔ::uapA-GFP thiAp-Anlsb6::AFpyrG nkuAΔ::argB pyrG89 pyroA4*	this study
thiA$_p$-Anlsb6<br>GFP-PH^{PLCδ}	*thiAp-Anlsb6::AFpyrG [gpdA::GFP::PH^{PLCδ}]-argBmut::argB2 pyroA4*	this study
thiA$_p$-Anlsb6<br>hypB-mScarlet	*thiAp-Anlsb6::AFpyrG hypB$^{-(5xGA)}$mScarlet::AFriboB nkuAΔ::argB pyrG89 pyroA4 riboB2*	this study
thiA$_p$-Anlsb6<br>gga2-GFP	*thiAp-Anlsb6::AFriboB gga2$^{-(5xGA)}$GFP::AFpyrG nkuAΔ::argB pyrG89 pyroA4 riboB2*	this study
thiA$_p$-Anlsb6<br>arfA-GFP	*thiAp-Anlsb6::AFriboB arfA$^{-(5xGA)}$GFP::AFpyrG nkuAΔ::argB pyrG89 pyroA4 riboB2*	this study
thiA$_p$-Anlsb6<br>GFP-rabE	*thiAp-Anlsb6::AFriboB GFP-RabE::AFpyrG nkuAΔ:argB pyrG89 pyroA4 riboB2*	this study
thiA$_p$-Anlsb6<br>ap1σ-GFP	*thiAp-Anlsb6::AFriboB ap1σ-(5xGA)GFP::AFpyrG nkuAΔ::argB pyrG89 riboB2 pyroA4*	this study
thiA$_p$-Anlsb6<br>GFP-rabE<br>mCherry-sedV	*thiAp-Anlsb6::AFriboB GFP-RabE::AFpyrG pyroA4::[pyroA::gpdAmp-mCherry-sedV] nkuAΔ:argB inoB2*	this study
thiA$_p$-Anlsb6<br>GFP-rabE<br>ap1σ-mRFP	*thiAp-Anlsb6::AFpyroA ap1σ$^{-(5xGA)}$mRFP::AFpyrG GFP-RabE::AFpyrG pyroA4*	this study
thiA$_p$-Anlsb6<br>mRFP-PH^{OSBP}<br>arfA-GFP	*thiAp-Anlsb6::AFriboB arfA$^{-(5xGA)}$GFP::AFpyrG [pyroA-gpdAm-mRFP-PH^{OSBP}]pyroA4 nkuAΔ::argB pyrG89 pyroA4 riboB2*	this study
thiA$_p$-Anlsb6<br>hypB- mScarlet<br>GFP-chsB	*GFP-chsB::AFpyrG thiAp-Anlsb6::AFpyrG hypB$^{-(5xGA)}$mScarlet::AFriboB nkuAΔ::argB pyroA4 riboB2 pantoB100*	this study
GFP-chsB<br>thiA$_p$-rabC	*GFP-chsB::AFpyrG thiAp-RabC::AFriboB nkuAΔ::argB pyrG89 pyroA4 riboB2*	this study
GFP-chsB<br>thiA$_p$-rabA	*GFP-chsB::AFpyrG thiAp-RabA::AFriboB nkuAΔ::argB pyrG89 pyroA4 riboB2*	this study
GFP-chsB<br>rabBΔ	*GFP-chsB::AFpyrG rabBΔ::AFpyroA nkuAΔ::argB pyrG89 pyroA4 riboB2*	this study
GFP-chsB<br>thiA$_p$-rabE	*GFP-chsB::AFpyrG thiAp-RabE::AFriboB nkuAΔ::argB pyrG89 pyroA4 riboB2*	this study
thiA$_p$-Anlsb6<br>mRFP-rabA<br>GFP-chsB	*GFP-chsB::AFpyrG thiAp-Anlsb6::AFpyrG gpdApm-mRFP-rabA::AFriboB nkuAΔ::argB pyrG89 pyroA4 riboB2 pantoB100*	this study
thiA$_p$-Anlsb6<br>mRFP-rabB<br>GFP-chsB	*GFP-chsB::AFpyrG thiAp-Anlsb6::AFpyrG gpdApm-mRFP-rabB-panB nkuAΔ::argB pyrG89 pyroA4 riboB2 pantoB100*	this study

thiA$_p$-Anlsb6<br>rabBΔ<br>GFP-chsB	*GFP-chsB::AFpyrG thiAp-Anlsb6::AFpyrG rabBΔ::AFpyroA nkuAΔ::argB pyroA4 riboB2 pantoB100*	this study
thiA$_p$-Anlsb6<br>thiA$_p$-rabA<br>GFP-chsB	*GFP-chsB::AFpyrG thiAp-Anlsb6::AFpyrG thiAp-rabA::AFriboB nkuAΔ::argB pyroA4 riboB2 pantoB100*	this study
thiA$_p$-Anlsb6<br>rabBΔ<br>mCherry-synA	*AFpyG-mcherry-synA thiAp-Anlsb6::AFpyrG rabBΔ::AFpyroA nkuAΔ::argB pyroA4*	this study
GFP-chsB<br>rabBΔ<br>thiA$_p$-rabA	*GFP-chsB::AFpyrG rabBΔ::AFpyroA thiAp-rabA::AFriboB nkuAΔ::argB pyrG89 pyroA4 riboB2*	this study
thiA$_p$-Anlsb6<br>GFP-chsB rabBΔ<br>thiA$_p$-rabA	*thiAp-Anlsb6::AFpyrG GFP-chsB::AFpyrG rabBΔ::AFpyroA thiAp-rabA::AFriboB nkuAΔ::argB pyroA4 riboB2 pantoB100*	this study
thiA$_p$-Anlsb6<br>GFP-chsB rabBΔ<br>thiA$_p$-rabC	*thiAp-Anlsb6::AFpyrG GFP-chsB::AFpyrG rabBΔ::AFpyroA thiAp-rabC::AFriboB nkuAΔ::argB pyroA4 riboB2 pantoB100*	this study
Anlsb6$_p$-lsb6	*Anlsb6p-lsb6-panB thiAp-Anlsb6::AFpyrG GFP-chsB::AFpyrG pyroA4 pabaA1 pantoB100*	this study
gpdA$_p$-lsb6	*gpdAp-lsb6-panB thiAp-Anlsb6::AFpyrG GFP-chsB::AFpyrG pyroA4 pabaA1 pantoB100*	this study
Anlsb6$_p$-pik1	*Anlsb6p-pik1-panB thiAp-Anlsb6::AFpyrG GFP-chsB::AFpyrG pyroA4 pabaA1 pantoB100*	this study
gpdA$_p$-pik1	*gpdAp-pik1-panB thiAp-Anlsb6::AFpyrG GFP-chsB::AFpyrG pyroA4 pabaA1 pantoB100*	this study
S288C	*S288C: MATa his3Δ1 leu2Δ0 met15Δ0 ura3Δ0*	Euroscarf
TSA116	*Y40675/TSA116: BY4741; MATa ura3Δ0 leu2Δ0 his3Δ1 met15Δ0 pik1-139:kanMX*	Euroscarf
pik1Δ	*BY4741; MATa ura3Δ0 leu2Δ0 his3Δ1 met15Δ0 pik1-139:kanMX PCJ313 (CEN-ARS LEU2 HIS3 MET25)*	this study
pik1Δ-pFL038	*BY4741; MATa ura3Δ0 leu2Δ0 his3Δ1 met15Δ0 pik1-139:kanMX PCJ313 (CEN-ARS LEU2 HIS3 MET25) pFL038-ura*	this study
pik1Δ-pCG563	*BY4741; MATa ura3Δ0 leu2Δ0 his3Δ1 met15Δ0 pik1-139:kanMX PCJ313 (CEN-ARS LEU2 HIS3 MET25) pCG563-ura*	this study
pik1$_p$-Anlsb6	*BY4741; MATa ura3Δ0 leu2Δ0 his3Δ1 met15Δ0 pik1-139:kanMX PCJ313 (CEN-ARS LEU2 HIS3 MET25) pFL038-pik1p-Anlsb6-ura*	this study
gal1$_p$-Anlsb6	*BY4741; MATa ura3Δ0 leu2Δ0 his3Δ1 met15Δ0 pik1-139:kanMX PCJ313 (CEN-ARS LEU2 HIS3 MET25) pCJ563- gal1p-Anlsb6-ura*	this study

References to Supplementary Table 1

1. Araujo-Bazán, L., Peñalva, M.A. & Espeso, E.A. Preferential localization of the endocytic internalization machinery to hyphal tips underlies polarization of the actin cytoskeleton in *Aspergillus nidulans*. *Mol. Microbiol.* **67,** 891-905. (2008) doi: 10.1111/j.1365-2958.2007.06102.x.
2. Dimou, S., Martzoukou, O., Dionysopoulou, M., Bouris, V., Amillis, S. & Diallinas, G. Translocation of nutrient transporters to cell membrane via Golgi bypass in *Aspergillus nidulans*. *EMBO Rep.* **21,** e49929. (2020). doi: 10.15252/embr.201949929.
3. Evangelinos, M., Martzoukou, O., Chorozian, K., Amillis, S. & Diallinas, G. BsdA(Bsd2) -dependent vacuolar turnover of a misfolded version of the UapA

transporter along the secretory pathway: prominent role of selective autophagy. *Mol. Microbiol.* **100,** 893-911. (2016). doi: 10.1111/mmi.13358.

4. Martzoukou, O., Amillis, S., Zervakou, A., Christoforidis, S. & Diallinas, G. The AP-2 complex has a specialized clathrin-independent role in apical endocytosis and polar growth in fungi. *Elife.* **6,** e20083. (2017). doi: 10.7554/eLife.20083
5. Martzoukou, O., Diallinas, G. & Amillis, S. Secretory Vesicle Polar Sorting, Endosome Recycling and Cytoskeleton Organization Require the AP-1 Complex in *Aspergillus nidulans*. *Genetics.* **209,** 1121-1138. (2018). doi: 10.1534/genetics.118.301240.
6. Nayak, T., Szewczyk, E., Oakley, C.E., Osmani, A., Ukil, L., Murray, S.L., Hynes, M.J., Osmani, S.A. & Oakley, B.R. A versatile and efficient gene-targeting system for *Aspergillus nidulans. Genetics*. **172,** 1557-1566. (2006). doi: 10.1534/genetics.105.052563.
7. Pantazopoulou A, Peñalva MA. Organization and dynamics of the Aspergillus nidulans Golgi during apical extension and mitosis. Mol Biol Cell. 2009 Oct;20(20):4335-47. doi: 10.1091/mbc.e09-03-0254.
8. Pantazopoulou, A. & Peñalva, M.A. Characterization of *Aspergillus nidulans* RabC/Rab6. *Traffic.* **12,** 386-406. (2011). doi: 10.1111/j.1600-0854.2011.01164.x.
9. Pantazopoulou, A. & Peñalva, M.A. Organization and dynamics of the *Aspergillus nidulans* Golgi during apical extension and mitosis. *Mol. Biol. Cell.* **20,** 4335-4347. (2009). doi: 10.1091/mbc.e09-03-0254.
10. Schultzhaus, Z., Yan, H. & Shaw, B.D. *Aspergillus nidulans* flippase DnfA is cargo of the endocytic collar and plays complementary roles in growth and phosphatidylserine asymmetry with another flippase, DnfB. *Mol. Microbiol.* **97,** 18-32. (2015). doi: 10.1111/mmi.13019.
11. Taheri-Talesh, N., Horio, T., Araujo-Bazán, L., Dou, X., Espeso, E.A., Peñalva, M.A., Osmani, S.A. & Oakley, B.R. The tip growth apparatus of *Aspergillus nidulans. Mol. Biol. Cell.* **19,** 1439-1449. (2008). doi: 10.1091/mbc.e07-05-0464.

Supplementary Table 2. Oligonucleotides used in this study.

	**Sequence 5'- 3'**
**pGEM-AnLsb6-(5xGA)GFP::AFpyrG/pGEM-GFP-AnLsb6::AFpyrG/ pGEM-GFP-AnLsb6::AFriboB**	
Anlsb6 5 ApaI F	CGCGGGGCCCGTCTCGAAGGGTATCTGTCAGCCTG
Anlsb6 5 SpeI R	CGCGACTAGTGAAGACTGCCAGACTTTCGGCTCC
Anlsb6 ORF SpeI F	CGCGACTAGTATGCCGAAAAACAATCGACCCGC
Anlsb6 ORF NotI R	CGCGGCGGCCGCGCCATGACAGACTGTTGTCAATGG
Anlsb6 ORF SphI F	CGCGCATGCGGGATCGAAGAAAAGCTTGGATGG
Anlsb6 ORFns SpeI R	CGCGACTAGTGCACCAAGTAAAAACAGGATTTTTACTC
GAGFP SpeI F	GCGCACTAGTGGAGCTGGTGCAGGCGCTGGAG
AFpyrG SpeI R	CGCGACTAGTCTGTCTGAGAGGAGGCACTGATG
AFriboB SpeI F	CGCGACTAGTAAGCTTGATATCACAATCAGCTTTTC
**pGEM-thiAp-Ansb6::AFpyrG/ pGEM-thiAp-Anlsb6::AFriboB/pGEM-thiAp-Anlsb6::AFpyroA/pGEM-thiApFLAG-Anlsb6::AFpyrG**	
Anlsb6 5 ApaI F	CGCGGGGCCCGTCTCGAAGGGTATCTGTCAGCCTG
Anlsb6 5 SpeI R	CGCGACTAGTGAAGACTGCCAGACTTTCGGCTCC
Anlsb6 3 SpeI F	CGCGACTAGTCGCTGGGCTTTCCATATCACCGG
Anlsb6 3 NotI R	CGCGGCGGCCGCCTCCAAGTTCGCTGTTCCCCACAG
thiA SpeI F	CGCGACTAGTCGACCTGGCACCTACAGAAGAATCC
thiA XbaI R	CGCGTCTAGAGTTGACTCAGTTCAATGGTTCGAC
thiA FLAG R	CGCGACTAGTCGCGACTAGTCTTGTCATCGTCGTCCTTGTAGTCCATGTTGACTCAGTTCAATGGTTC
AFpyrG SpeI F	CGCGACTAGTGCCTCAAACAATGCTCTTCACCC
AFpyrG SpeI R	CGCGACTAGTCTGTCTGAGAGGAGGCACTGATG
AFriboB SpeI F	CGCGACTAGTAAGCTTGATATCACAATCAGCTTTTC
AFriboB SpeI R	CGCGTCTAGACCCGGGCTGCAGGAATTCGATAAG
AFpyroA SpeI F	CGCGACTAGTGGACATCAGATGCTGGATTAC
AFpyroA SpeI R	CGCGACTAGTGCGAGTGTCTACATAATGAAGG
**pGEM-arfA-(5xGA)GFP::AFpyrG**	
arfA 5 ApaI F	CGCGGGGCCCCGGCGGCTGAATTTCTCGACCTG
arfA ORFns SpeI R	CGCGACTAGTGTCGCGGCCAGTCTTCCGCAG
arfA 3 SpeI F	CGCGACTAGTGCGGTAGTTGTGATGAATGGATAG
arfA 3 NotI R	CGCGGCGGCCGCCCTCATCCACAGCATCCGCAAAGAC
GAGFP SpeI F	GCGCACTAGTGGAGCTGGTGCAGGCGCTGGAG
AFpyrG SpeI R	CGCGACTAGTCTGTCTGAGAGGAGGCACTGATG
**pGEM-hypB-(5xGA)mScarlet::AFriboB**	
hypB ORF ApaI F	CGCGGGGCCCCTAGGGCCTTGACATACCTTTTCG
hypB ORFns SpeI R	CGCGACTAGTGCGCCGGCCGACGCTATGCTTGCGAG
hypB 3 SpeI F	CGCGACTAGTCCGCTTTAGACGGCTCCTATATTCAG
hypB 3 NotI R	CGCGGCGGCCGCGAGAAGCGGATGCGAGTTGCCTGAG
GAmScar SpeI F	CGCGACTAGTGGAGCAGGTGCTGGTGCTGGTGCTGGAGCAGTGAGCAAGGGCGAGGCAGTG
mScar XbaI R	CGCGTCTAGATTACTTGTACAGCTCGTCCATGCCGCC
AFpyrG SpeI F	CGCGACTAGTGCCTCAAACAATGCTCTTCACCC
AFpyrG SpeI R	CGCGACTAGTCTGTCTGAGAGGAGGCACTGATG
**pGEM-gga2-(5xGA)GFP::AFpyrG**	
gga2 ORF ApaI F	CGCGGGGCCCGTCAGAACTGTGTTGACCTGGGTTG
gga2 ORF SpeI NS R	CGCGACTAGTTGCAATGCCCAGCGGTGGTACC
gga2 3 SpeI F2	CGCGACTAGTCGCCACTCACTCGGCCACGATTAG
gga2 3 NotI R2	CGCGGCGGCCGCCCAGAGCGTTCCAATTCCACCCAG
GAGFP SpeI F F	GCGCACTAGTGGAGCTGGTGCAGGCGCTGGAG
AFpyrG SpeI F R	CGCGACTAGTCTGTCTGAGAGGAGGCACTGATG
**pGEM-Anlsb6p-lsb6-panB/pGEM-gpdAp-lsb6-panB**	
Anlsb6 pr ApaI F	CGCGGGGCCCGCGCTTGATCAGTGATGAACCGTTC
Anlsb6 5 SpeI R2	CGCGACTAGTGAAATAATATTGAAAGGGGCGTGAATGAAAGGC
Lsb6 SpeI F	CGCGACTAGTATGAGTAACGAAGCTTACCAGCATGATC
Lsb6 NotI R	CGCGGCGGCCGCTCAACACCAGGTGAATACGGGGGTG

**pGEM-Anlsb6p-pik1-panB/ pGEM-gpdAp-pik1-panB**	
Anlsb6 pr ApaI F	CGCGGGGCCCGCGCTTGATCAGTGATGAACCGTTC
Anlsb6 5 SpeI R2	CGCGACTAGTGAAATAATATTGAAAGGGGCGTGAATGAAAGGC
Pik1 XbaI F	CGCGTCTAGAATGCATAAAGCATCCAGTTCAAAGAAAAGC
Pik1 NotI R	CGCGGCGGCCGCTCAGCTATATATACCCTGTGTAATAAGTTGAAATTGG
**pFL038-pik1p-Anlsb6-ura/pCJ563- gal1p-Anlsb6-ura**	
Pik1 pr F	TCCCAGTCACGACGTTGTAAAACGACGGCCAGTGAATTCGAGCTCTTGCATAGTCACAGA
Pik1 R	CACACAGGAAACAGCTATGACCATGATTACGCCAAGCTTGCATGCGATAATAATTTATATGTATGGTTAAT
Pik1 ups F	CTCCTTGATTCTTTTAGCCG
Pik1 down R	CGAGCTCAAGATTCCGAAAG
Anlsb6 PIK1pr F	TTCATTGCGAGCGTTCGTTTTCATTAAGCATTCGTTCTCTTCACTATGCCGAAAAACAATCGACC
Anlsb6 R	TTGCAAATTGTTTTTCCAGTTCTGTCACAGCTATTTTCCTTCTCAGCACCAAGTAAAAACAGGAT
Anlsb6 GAL1 F	TGTTAATATACCTCTATACTTTAACGTCAAGGAGAAAAAACTATAATGCCGAAAAACAATCGACC
Anlsb6 R	TTGCAAATTGTTTTTCCAGTTCTGTCACAGCTATTTTCCTTCTCAGCACCAAGTAAAAACAGGAT
