# Supplementary material for: A type II phosphatidylinositol-4-kinase coordinates sorting of cargo polarizing by endocytic recycling
Source: Commun Biol. 2024 Jul 12;7:855. doi: 10.1038/s42003-024-06553-3 (PMC11245547; doi:10.1038/s42003-024-06553-3)
Supplement: Supplementary file 2 — Description of Additional Supplementary Files [file 42003_2024_6553_MOESM2_ESM.pdf]

Description of Additional Supplementary Files

File name: Supplementary Data 1

Description: The source data behind the graph in Figure 1c.

File name: Supplementary Data 2

Description: The source data behind the graph in Figure 2a.

File name: Supplementary Data 3

Description: The source data behind the graph in Figure 3a.

File name: Supplementary Data 4

Description: The source data behind the graph in Supplementary Figure 4.

File name: Supplementary Data 5

Description: The source data behind the graph in Figure 3d.

File name: Supplementary Data 6

Description: The source data behind the graph in Supplementary Figure 5.

File name: Supplementary Data 7

Description: The source data behind the graph in Supplementary Figure 6.

File name: Supplementary Data 8

Description: The source data behind the graph in Figure 4c.
